# Alternative Pathway Dysregulation and the Conundrum of Complement Activation by IgG4 Immune Complexes in Membranous Nephropathy

**DOI:** 10.3389/fimmu.2016.00157

**Published:** 2016-04-25

**Authors:** Dorin-Bogdan Borza

**Affiliations:** ^1^Department of Microbiology and Immunology, Meharry Medical College, Nashville, TN, USA

**Keywords:** membranous nephropathy, IgG4, complement, alternative pathway, factor H, heparan sulfate, glomerular basement membrane

## Abstract

Membranous nephropathy (MN), a major cause of nephrotic syndrome, is a non-inflammatory immune kidney disease mediated by IgG antibodies that form glomerular subepithelial immune complexes. In primary MN, autoantibodies target proteins expressed on the podocyte surface, often phospholipase A2 receptor (PLA2R1). Pathology is driven by complement activation, leading to podocyte injury and proteinuria. This article overviews the mechanisms of complement activation and regulation in MN, addressing the paradox that anti-PLA2R1 and other antibodies causing primary MN are predominantly (but not exclusively) IgG4, an IgG subclass that does not fix complement. Besides immune complexes, alterations of the glomerular basement membrane (GBM) in MN may lead to impaired regulation of the alternative pathway (AP). The AP amplifies complement activation on surfaces insufficiently protected by complement regulatory proteins. Whereas podocytes are protected by cell-bound regulators, the GBM must recruit plasma factor H, which inhibits the AP on host surfaces carrying certain polyanions, such as heparan sulfate (HS) chains. Because HS chains present in the normal GBM are lost in MN, we posit that the local complement regulation by factor H may be impaired as a result. Thus, the loss of GBM HS in MN creates a micro-environment that promotes local amplification of complement activation, which in turn may be initiated *via* the classical or lectin pathways by subsets of IgG in immune complexes. A detailed understanding of the mechanisms of complement activation and dysregulation in MN is important for designing more effective therapies.

## Introduction

One of the major causes of idiopathic nephrotic syndrome in adults, membranous nephropathy (MN) is an immune kidney disease is mediated by IgG antibodies that form glomerular subepithelial immune complexes (1). Histologic hallmarks of MN are granular capillary loop deposits containing antigen, IgG, and complement, an expansion of the glomerular basement membrane (GBM), subepithelial electron-dense deposits, and podocyte foot process effacement, but little glomerular inflammation. Clinically, MN presents as proteinuria and often nephrotic syndrome. The natural course of the disease is variable. While some patients spontaneously remit, up to 40% of MN patients progress to end-stage renal disease over a period of 5–10 years. Another one-third of MN patients have persistent nephrotic syndrome, often with secondary complications of hyperlipidemia and hypercoagulability (2).

The current paradigm for the pathogenesis of MN has emerged from studies of Heymann nephritis, a rat model closely recapitulating human MN (3). The central pathogenic events are the formation of subepithelial immune complexes, which activate complement, thereby causing complement-mediated podocytes injury and proteinuria. This article overviews the mechanisms of complement activation and regulation in MN. The author further posits that complement activation in MN is exacerbated by GBM alterations, which impair normal complement regulation.

## Immune Complexes, Target Antigens, and Antibodies Mediating MN

Membranous nephropathy has a heterogeneous etiology. About 25% of cases, classified as secondary MN, are associated with autoimmune disease (most often lupus nephritis), infections, toxins, drugs, or malignancy. More common is primary (idiopathic) MN, now understood as an organ-specific autoimmune disease. Subepithelial immune complexes may form by several mechanisms (4). In secondary MN, immune complexes typically form when “planted antigens” extrinsic to the glomerulus become lodged in the subepithelial space and subsequently bind IgG antibodies. The target antigens are not always known. An example is cationic bovine serum albumin (presumably of dietary origin) in pediatric MN (5).

Relevant to primary MN, in the paradigm established in rat Heymann nephritis models, an intrinsic antigen located on the podocyte cell surface binds antibodies, forming *in situ* immune complexes, which are shed subepithelially. In rats, megalin is the major target of antibodies induced by immunization with crude Fx1A antigen (6). In human disease, the first podocyte antigen identified is neutral endopeptidase (NEP), targeted in rare forms of alloimmune MN (7, 8). NEP-deficient mothers who are allo-immunized during a previous miscarriage produce anti-NEP alloantibodies that cross the placenta and bind to NEP in the fetal kidneys, causing antenatal MN.

Primary MN is mediated by IgG autoantibodies targeting proteins on the podocyte cell surface. Phospholipase A2 receptor (PLA2R1), a glycoprotein from the mannose receptor family, is targeted by autoantibodies in ~70% of patients with primary MN (9). Another 3–5% of patients with primary MN have autoantibodies targeting thrombospondin type-1 domain-containing 7A (THSD7A), another podocytes glycoprotein (10). Additional autoantibodies to proteins expressed intracellularly by podocytes (aldose reductase, manganese superoxide dismutase, and alpha-enolase), possibly generated after the initial injury by inter-molecular epitope spreading, are variably present in MN (11, 12); their pathogenic significance remains uncertain.

How antibodies causing MN mediate glomerular injury is incompletely understood. Human IgG comprises four subclasses with different effector ability (13). Most often in primary MN (but rarely in secondary MN), IgG4 is the major subclass of antibodies forming subepithelial immune complexes. IgG4 antibodies are non-inflammatory because they undergo dynamic Fab arm exchange, swapping half-molecules to form bispecific, functionally monovalent IgG4 (14). Relevant to the focus of this article, IgG4 does not activate complement (15). This poses the conundrum of how complement is activated in primary MN.

## Complement Activation in MN

The complement system is a component of the innate immunity, which provides host defense against pathogens and is also important for the clearance of immune complexes and damaged cells and for immunoregulation (16). However, excessive complement activation or insufficient regulation causes tissue injury in many autoimmune or inflammatory diseases (17). Kidney glomerulus is particularly sensitive to complement-mediated injury (18).

### Overview of the Complement Cascade and Effector Mechanisms

Activation of the complement cascade is initiated by three pathways (classical, lectin, and alternative) converging toward the generation of C3 convertases, which cleave C3 into C3a and C3b. Addition of C3b to C3 convertases generates C5 convertases, which cleave C5 into C5a and C5b, activating the terminal complement pathway. C5b sequentially binds C6, C7, C8 and C9, forming C5b–9. Effector molecules produced by complement activation include anaphylatoxins (C3a, C5a) that recruit and activate inflammatory cells, opsonins (C3b, iC3b) that bind to target surfaces and promote phagocytosis, and the membrane attack complex (C5b–9), which lyses cells.

Complement activation plays a key role in the pathogenesis of MN (3, 19, 20). In human and experimental MN, C3 and C5b–9 commonly accompany IgG in subepithelial deposits (21, 22). C3d, a stable product of C3b breakdown, is found in glomerular deposits of all MN patients, while C3c staining (detecting C3b/iC3b) may be absent in patients with less proteinuria (23), possibly reflecting inactive disease. In this regard, glomerular C3c staining indicates ongoing complement activation while C3d is a marker of past complement activation (24). The urinary excretion of C3dg and C5b–9 correlates with disease activity in primary MN (25–27). In Heymann nephritis, proteinuria can be prevented by the depletion of C3 and also of C6 (28, 29), the latter implicating podocyte injury by C5b–9 as a major effector mechanism, as first shown in perfused rat glomeruli (30). Sublethal injury by C5b–9 triggers maladaptive changes in podocytes that disrupt the glomerular filtration barrier and cause proteinuria, reviewed in detail elsewhere (31–33).

### Classical Pathway

The *classical pathway* is initiated when C1q binds to immune complexes containing IgM or certain IgG subclasses. Consequent activation of C1r and C1s cleaves C4, eventually forming C4b2b, the C3 convertase of the classical and lectin pathways (LPs). Among human IgG subclasses, IgG3 and IgG1 strongly bind C1q and activate complement, while IgG4 does not (13). In antenatal alloimmune MN, complement-fixing IgG1 anti-NEP alloantibodies are associated with severe proteinuria, whereas IgG4 anti-NEP cause limited disease (34). Likewise, in active and passive Heymann nephritis, only complement-fixing subclasses (sheep γ1, rat IgG2b) induce proteinuria (28, 35).

Paradoxically, in primary MN, subepithelial immune complexes contain predominantly (but not exclusively) IgG4 (36), which neither binds C1q nor activates complement. C1q staining in primary MN is absent (37) or weak, as detected using more sensitive staining (21). In patient sera, quantitative immunoassays of anti-PLA2R subclasses also show a prevalence of IgG4, which on average comprises ~50% of anti-PLA2R IgG (38). Smaller proportions of IgG1 (~9%) and IgG3 (~6%) are also found, which may be sufficient to activate the classical pathway, albeit inefficiently.

The role of IgG4 antibodies in MN – whether they are protective or pathogenic – remains a conundrum. In 5–10% of patients seropositive for anti-PLA2R1, IgG4 autoantibodies are negative (39). A unique case of recurrent MN features monoclonal IgG3-kappa anti-PLA2R1, associated with glomerular C1q deposition (40). Therefore, IgG4 antibodies are not absolutely required for primary MN. Interestingly, IgG1 is prevalent in the earlier stages of primary MN, as in secondary MN, while IgG4 staining (inversely correlated with C1q) prevails at later stages of primary MN (41). This suggests that IgG autoantibodies undergo a subclass switch from IgG1 to IgG4 during disease progression, as a temporal model of IgG function proposes (42). If so, the classical pathway may be more important in early MN, while other pathways become dominant as disease progresses (43).

### Lectin Pathway

The LP is initiated when mannan-binding lectin (MBL) or ficolins bind to patterns of carbohydrates present on pathogens or damaged self, activating MBL-associated serine proteases to produce C2b4b. MBL binds to “G0” glycoforms of IgG that lack terminal sialic and galactose residues on the conserved N-glycan in the Fc region, activating the LP (44). IgG–G0 glycoforms comprise ~25% of all IgG in normal human sera, but are increased in some autoimmune diseases (45). Glomerular deposition of MBL occurs in some MN patients (21, 46), suggestive of the LP activation. This is consistent with preliminary studies reporting that affinity-purified anti-PLA2R1 IgG4 autoantibodies bind MBL, promoting C4 deposition (47). Further studies extending these investigations to other IgG subclasses and other pathways are needed to clarify the contribution of the LP to overall complement activation by anti-PLA2R1 autoantibodies.

### Alternative Pathway

**Figure 1:**
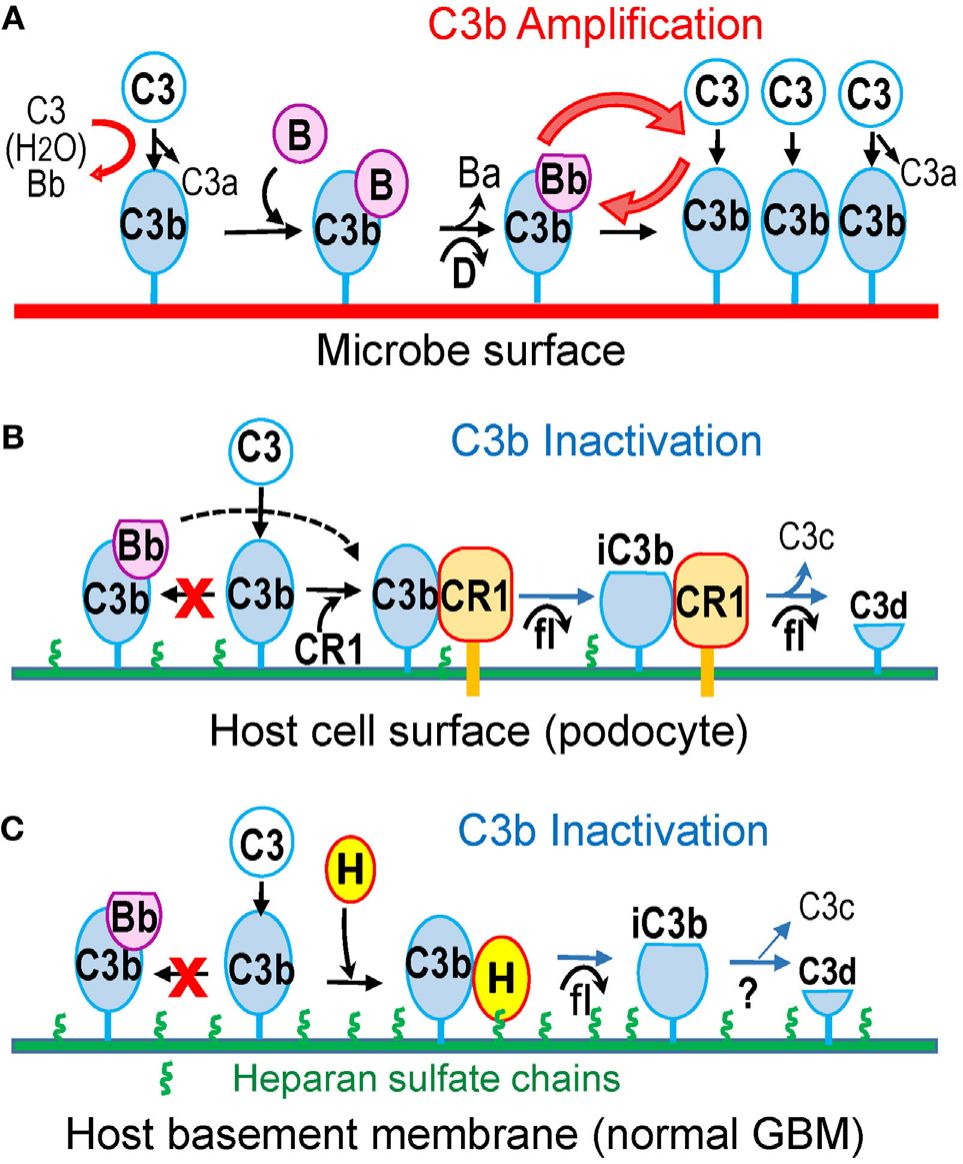
**Alternative pathway amplifies complement activation on pathogen surfaces but not on host surfaces protected by complement regulatory proteins (CRP)**. **(A)** C3b is continuously generated in fluid phase due to tick-over. C3b deposited on complement-activating surfaces (such as microbes) binds factor B, which is cleaved by factor D to form the C3bBb convertase, thus amplifying C3b generation. **(B)** On the surface of host cells, such as podocytes, membrane-bound CRPs (illustrated by CR1) catalyze factor I-mediated proteolytic inactivation of C3b to iC3b and C3d. CR1 also accelerates the decay of C3bBb convertase, if present (dotted line). **(C)** The extracellular matrix, such as the normal GBM, contains heparan sulfate chains (green), which recruit factor H from plasma to inactivate surface-bound C3b in a manner similar to cell-bound CRPs.

The alternative pathway (AP) is constitutively active at low levels. Slow spontaneous hydrolysis of the thioester bond of C3 (“tickover”) generates C3(H_2_O), which in the presence of factors B and D produces C3(H_2_O)Bb, the initial C3 convertase of the AP. This cleaves C3 to C3b, unmasking the reactive thioester, which allows C3b to attach covalently to surfaces. Unless inactivated by complement regulatory proteins (next section), surface-bound C3b binds factor B, allowing cleavage by factor D to form C3bBb, the major C3 convertase of the AP. C3bBb cleaves additional C3 molecules, generating more surface-bound C3b (Figure 1A). Through this positive feedback loop, the AP amplifies complement activation, even when C3b is initially produced by other pathways (48). Amplification is limited by the intrinsic instability of C3bBb, which decays when Bb dissociates irreversibly. Properdin stabilizes surface-bound C3bBb, significantly extending its half-life (49). In addition, properdin tethered to surface-bound C3b or tissue glycosaminoglycans may direct AP activation by providing a platform for C3bBb convertase assembly (50–52).

Glomerular deposition of factor B found in MN biopsies provides circumstantial evidence that the AP is activated in disease (21). Moreover, MN was reported in a patient with C4 deficiency (53), in which the classical and LPs are not functional. In preliminary studies, we evaluated the role of the AP in experimental MN in mice. Mice immunized with α3(IV) collagen NC1 domain develop subepithelial immune complexes and proteinuria, recapitulating clinical and morphologic hallmarks of MN (54, 55). In factor B^−/−^ mice, without a functional AP, glomerular C3c and C5b–9 deposition and proteinuria were prevented, while circulating and kidney-bound IgG were unchanged (56). These findings imply that the AP is required for complement activation and proteinuria in experimental MN.

## Complement Regulation in MN

Activation of complement, and in particular that of the AP, must be closely regulated to avoid damage to the host. This is achieved by cell-associated and fluid-phase complement regulatory proteins (CRP).

### Cell-Bound Complement Regulators

Glomerular cells express several membrane-bound CRPs, which inactivate the C3/C5 convertases of all pathways in the immediate proximity of cell surfaces (20). These include decay-accelerating factor (DAF, CD55), which inhibits complement activation by accelerating the dissociation of all convertases, membrane cofactor protein (MCP, CD46), which catalyzes the proteolytic inactivation of C3b/C4b by factor I, and complement receptor 1 (CR1, CD35), which has both decay-accelerating and cofactor activity (Figure 1B). In addition, cells are protected by membrane-bound CD59, which inhibits the formation of C5b–9.

Studies of experimental MN demonstrate the protective role of membrane-bound CRPs. In active Heymann nephritis, complement activation and proteinuria are dependent on the formation of function-blocking antibodies to Crry (the rodent equivalent of human CR1) and CD59, which are present in crude preparations of Fx1A antigen used to induce disease (57). Anti-Fx1A antibodies used to induce passive Heymann nephritis also contain anti-Crry and anti-CD59 antibodies, which *in vitro* inhibit complement regulation on rat podocytes, allowing complement activation *via* the AP (58, 59). Although autoantibodies to podocyte CRPs have not been described in human MN, staining for CR1 on podocytes is decreased in MN and other glomerular diseases (60). An acquired loss of CR1 may increase podocyte susceptibility to complement-mediated attack.

### Fluid Phase Complement Regulators: Factor H

The major regulator of the AP in the fluid phase is factor H, an abundant plasma glycoprotein is composed of 20 domains named short consensus repeats (SCRs). Factor H inhibits the AP by accelerating the decay of the C3bBb convertase and by catalyzing proteolytic inactivation of C3b to iC3b by factor I (61, 62). These complement regulatory activities are mediated by the amino-terminal SCR1–4 of factor H (63), which are necessary and sufficient to inhibit the AP in the fluid phase.

Factor H also inhibits the AP on host surfaces carrying certain polyanions as markers of self. Factor H has two distinct heparin-/glycosaminoglycan-binding sites, located in the SCR7 and SCR19–20 (64), which recognize heparan sulfate (HS) chains in a tissue-specific manner (65). In addition, SCR20 binds specific sialic acid structures (66). Recognition of these host-specific polyanions enables factor H to discriminate between self and pathogen surfaces. Surface polyanions increase the affinity of factor H for surface-bound C3b, exposing its complement regulatory domains to inactivate C3b (67–69). Consequently, host surfaces coated by HS (or sialic acid) are complement non-activators because they recruit factor H effectively to inhibit the AP (70–72). In contrast, surfaces lacking these polyanions do not bind factor H, allowing complement activation and amplification. Impaired attachment of factor H to polyanions on glomerular endothelial cells, as a result of mutations or inhibitory autoantibodies, causes atypical hemolytic uremic syndrome, even though AP regulation in plasma is normal (73–76).

### Loss of Glomerular Heparan Sulfate in MN May Impair Local AP Regulation

Lacking protection from cell-bound CRPs (in contrast to podocytes), the GBM must recruit plasma factor H for local AP regulation. Putative ligands are HS chains attached to agrin core protein, which are particularly abundant in the normal GBM (77). Indeed, factor H (as well as its carboxyl-terminal domains SCR19–20) binds to glomeruli in a manner that can be inhibited by heparin, suggesting interactions with glycosaminoglycans (78). In functional assays, HS from the eye Bruch’s membrane inhibits the AP (79). Similarly, glomerular HS proteoglycans may recruit factor H to locally inhibit the AP in the GBM (Figure 1C).

A striking loss of HS chains from the GBM (detected by staining with mAb JM403) occurs in human MN, while staining for agrin core protein is unaltered (80). The loss of GBM HS, correlated with complement deposition and albuminuria, is recapitulated in active and passive Heymann nephritis (81, 82). The underlying mechanism may be an upregulation of heparanase in glomeruli (83). Heparanase, a beta-d-endoglycosidase, is the only mammalian enzyme that degrades HS chains. Rarely expressed in normal tissues, heparanase is upregulated in various pathologic conditions (84). An upregulation of glomerular heparanase occurs in Heymann nephritis, which is prevented by C3 depletion (85). Increased glomerular staining and urinary excretion of heparanase occurs in human MN (86).

**Figure 2:**
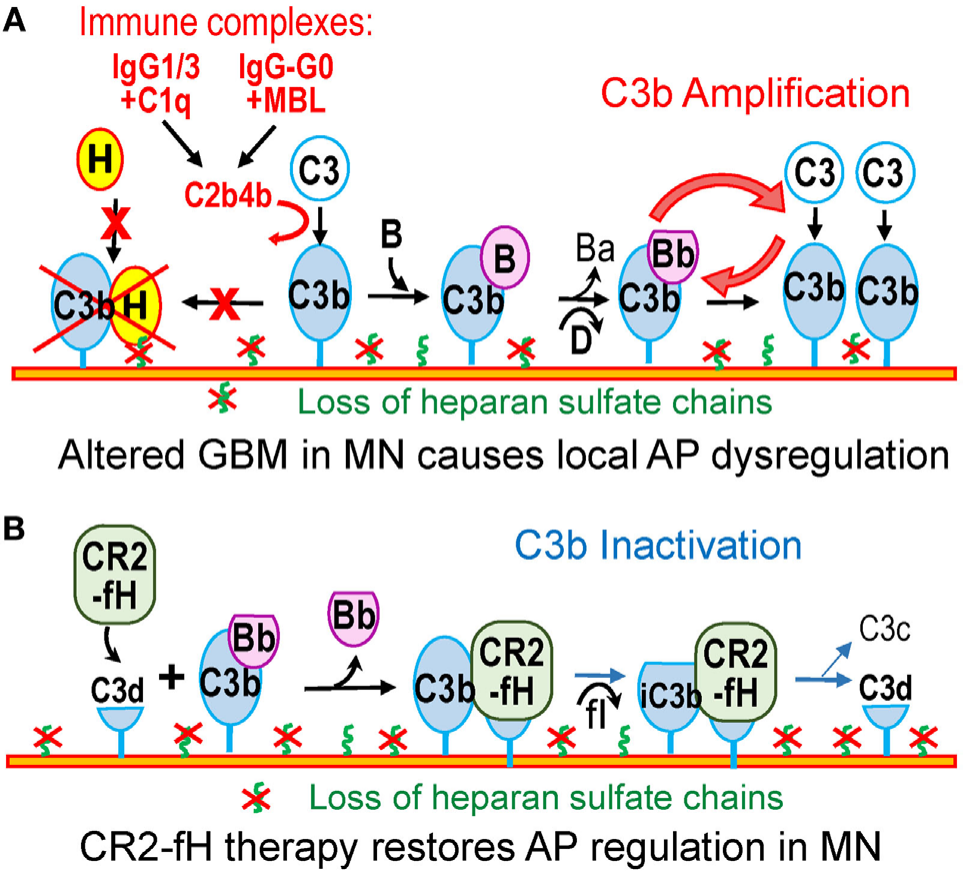
**Dysregulation of the alternative pathway in MN**. **(A)** In MN, subsets of IgG1/3 or IgG–G0 in subepithelial immune complexes may activate the classical or lectin pathway. C3b thus generated attaches to nearby targets in the GBM or on podocytes. The loss of heparan sulfate in the GBM in MN impairs recruitment of factor H and inactivation of C3b. As a result, the altered GBM resembles a pathogen surface that promotes C3b amplification. **(B)** Therapy with CR2–fH fusion protein overcomes the effects of the AP dysregulation in MN. CR2–fH is targeted at sites of complement activation in the GBM where iC3b or C3d are present, thus restoring the local inhibition of the AP.

We postulate that in MN, the acquired loss of glomerular HS chains would impair the ability of factor H to inactivate C3b deposited within the GBM (Figure 2A). Thus, alterations of the GBM composition in MN may lead to the local dysregulation of the AP. Similar to pathogen surfaces, the altered GBM would allow amplification of complement activation, which in turn may be initiated *via* the classical (or lectin) pathway by subsets of IgG1/IgG3 (or IgG–G0) in immune complexes. By itself, the loss of GBM HS appears insufficient to trigger sustained glomerular complement activation in the absence of glomerular immune complexes; for instance, glomerular C3c is absent in diabetic nephropathy despite the loss of GBM HS chains. Therefore, subepithelial immune complexes and the local AP dysregulation may both contribute to complement activation in MN, synergistically and by distinct mechanisms.

## Concluding Remarks: Complement as a Therapeutic Target in MN

More specific and effective therapies for treating MN remain a significant unmet need (87). Current therapies for MN rely on non-specific immunosuppression with cytotoxic agents or calcineurin inhibitors (along with low dose steroids), which have toxic side effects and are sometimes ineffective (88). A more specific intervention, the depletion of B cells by rituximab (an anti-CD20 monoclonal antibody) has yielded promising results in small studies, though not all patients respond (89–91). Even when immuno-suppressive therapies work, pathogenic antibodies persist for many months and cause additional injury. Clinical improvement is further delayed as the reduction of proteinuria lags behind immunological remission (disappearance of antibodies) by several months (92, 93). Complement inhibition may prevent further glomerular damage until pathogenic antibodies disappear.

There are few clinical studies of complement inhibitors in MN. Eculizumab is a humanized IgG2/IgG4 mAb that inhibits terminal complement activation by binding to C5 and preventing its cleavage. A randomized trial of eculizumab in MN did not show significant reduction in proteinuria after 16 weeks (94), perhaps because of an insufficient dosage regimen (20). Of note, blocking complement at the level of C5 does not affect the upstream C3 convertases. It is therefore possible that excessive glomerular C3 deposition may also interfere with the glomerular filtration function, independent of C5b–9 formation. In Cfh^−/−^ mice (a model of C3 glomerulopathy, a kidney disease caused by the AP dysregulation), the ablation of C5 improves survival and reduces glomerular inflammation (mediated by C5a), but does not reduce proteinuria or glomerular C3 staining (95).

Therapeutic inhibition of the AP has the potential to limit amplification of complement activation, which may be beneficial in MN. Supporting this concept are our preliminary studies showing that the genetic ablation of factor B in mice uncouples subepithelial immune complexes from glomerular C3 deposition and proteinuria in experimental MN (56). Effective agents for systemic inhibition of the AP *in vivo* include anti-factor B monoclonal antibodies and anti-sense oligonucleotides (96, 97).

Another attractive strategy would be to correct specifically the local dysregulation of the AP, which can be achieved by using complement inhibitors targeted to sites of complement activation (98). The poster child for this approach is CR2–fH, a fusion protein comprising a fragment of complement receptor 2 (CR2) that recognizes C3b breakdown products (iC3b, C3dg, and C3d), linked to the complement inhibitory domains of factor H (99). CR2–fH reduces complement-mediated kidney injury in mouse models lupus nephritis and C3 glomerulopathy (100, 101). Because C3b breakdown fragments are deposited in the glomeruli in MN, CR2–fH can bind at these sites to inhibit the AP even in the absence of HS chains (Figure 2B). In summary, a detailed understanding of the mechanisms of complement activation and dysregulation in MN is necessary to inform the design of safer, more effective, and specific therapies.

## Author Contributions

D-BB developed the concepts, wrote the initial draft, revised the article, and approved the final version for publication.

## Conflict of Interest Statement

The author declares that the research was conducted in the absence of any commercial or financial relationships that could be construed as a potential conflict of interest.
